# Detection of naturally acquired, strain-transcending antibodies against rosetting *Plasmodium falciparum* strains in humans

**DOI:** 10.1128/iai.00015-24

**Published:** 2024-06-06

**Authors:** Florence E. McLean, Yvonne Azasi, Cameron Sutherland, Emmanuel Toboh, Daniel Ansong, Tsiri Agbenyega, Gordon Awandare, J. Alexandra Rowe

**Affiliations:** 1 Institute of Immunology and Infection Research, School of Biological Sciences, University of Edinburgh, UK; 2 Manhyia District Hospital, Kumasi, Ghana; 3 Kwame Nkrumah University of Science and Technology, School of Medical Sciences, Kumasi, Ghana; Departments of Child Health and Medicine, Komfo Anokye Teaching Hospital, Kumasi, Ghana; 4 Malaria Research Centre, Agogo, Ghana; 5 West African Centre for Cell Biology of Infectious Pathogens, University of Ghana, Legon, Ghana

**Keywords:** severe malaria, PfEMP1, rosette formation, virulence, immunology, antibodies, epitopes

## Abstract

Strain-transcending antibodies against virulence-associated subsets of *P. falciparum* infected erythrocyte surface antigens could protect children from severe malaria. However, the evidence supporting the existence of such antibodies is incomplete and inconsistent. One subset of surface antigens associated with severe malaria, rosette-mediating *Plasmodium falciparum* Erythrocyte Membrane Protein one (PfEMP1) variants, cause infected erythrocytes to bind to uninfected erythrocytes to form clusters of cells (rosettes) that contribute to microvascular obstruction and pathology. Here, we tested plasma from 80 individuals living in malaria-endemic regions for IgG recognition of the surface of four *P. falciparum* rosetting strains using flow cytometry. Broadly-reactive plasma samples were then used in antibody elution experiments in which intact IgG was eluted from the surface of infected erythrocytes and transferred to heterologous rosetting strains to look for strain-transcending antibodies. We found that seroprevalence (percentage of positive plasma samples) against allopatric rosetting strains was high in adults (63%-93%) but lower in children (13%-48%). Strain-transcending antibodies were present in nine out of eleven eluted antibody experiments, with six of these recognising multiple heterologous rosetting parasite strains. One eluate had rosette disrupting activity against heterologous strains, suggesting PfEMP1 as the likely target of the strain-transcending antibodies. Naturally acquired strain-transcending antibodies to rosetting *P. falciparum* strains in humans have not been directly demonstrated previously. Their existence suggests that such antibodies could play a role in clinical protection and raises the possibility that conserved epitopes recognised by strain-transcending antibodies could be targeted therapeutically by monoclonal antibodies or vaccines.

## Introduction

During the blood stage of *Plasmodium falciparum* malaria, the parasite modifies the surface of its host erythrocyte by displaying variant surface antigens (VSAs) on the infected cell surface. These VSAs are derived from polymorphic protein families, the most well studied of which is *P. falciparum* erythrocyte membrane protein 1 (PfEMP1), which is a family of adhesion proteins that mediates sequestration of infected cells in the microvasculature (1). Other VSA families include the repetitive interspersed family (RIFIN), subtelomeric variant open reading frame (STEVOR) and surface-associated interspersed protein (SURFIN) families (2). Some PfEMP1 variants cause rosetting (3–7), where the infected erythrocyte binds to uninfected erythrocytes, and there is also some limited evidence for the involvement of RIFIN and STEVOR in rosetting interactions (8, 9). Rosetting enhances microvascular obstruction (10), and has been consistently associated with severe malaria in African children (11–14). For this reason, rosetting is considered a parasite virulence phenotype.

It has been hypothesised that antibodies which target VSAs (PfEMP1 in particular), are clinically protective (15, 16). In addition, the diversity of PfEMP1 variants, and the fact that each *P. falciparum* isolate encodes roughly 60 distinct variants, with minimal overlap between the PfEMP1 repertoires of different isolates (17), strongly suggests that PfEMP1 is an important immune target. As protection against severe malaria is acquired after just a few infections (18), naturally acquired strain-transcending antibodies against VSAs must exist if humoral responses to polymorphic parasite antigen families contribute to protection against severe malaria. Such strain-transcending antibodies, defined here as antibodies against the surface of live infected erythrocytes that recognise multiple genetically distinct *P. falciparum* strains, are thus targeting conserved rather than variant-specific epitopes.

Previous work shows that sera from semi-immune adults living in malaria endemic regions have broad reactivity with the infected erythrocyte surface of diverse parasite strains, as demonstrated by agglutination assays or flow cytometry (15, 16, 19, 20). This broad serological recognition could be due to the presence of strain-transcending antibodies, or could be due to acquisition of an extensive pool of variant-specific antibodies, and most serological studies do not differentiate between these two possibilities. A small number of studies have identified human strain-transcending monoclonal and polyclonal antibodies to VAR2CSA, the PfEMP1 variant that causes infected erythrocyte adhesion to placental syncytiotrophoblasts (21–23), and broadly strain-transcending antibodies to RIFIN variants have been described (24). The only study that has sought to identify human strain-transcending antibodies to rosetting parasites found only variant-specific responses (6). However, only three rosetting strains were studied (6), none of which had the dual rosetting and IgM Fc-binding adhesion phenotype that is most strongly associated with severe malaria (25–27), and which can be targeted by strain-transcending antibodies against PfEMP1 raised in rabbits (7).

We hypothesised that people living in malaria-endemic regions acquire strain-transcending IgG antibodies against virulence-associated VSAs, and sought to test this hypothesis by first identifying plasma samples from malaria-exposed humans that recognise multiple parasite strains displaying the rosetting IgM Fc-binding phenotype, and then using the broadly-reactive plasma in IgG elution experiments to test for homologous and heterologous infected erythrocyte recognition.

## Results

### Detection of naturally acquired human IgG to rosetting P. falciparum strains

First, we investigated whether individuals living in malaria endemic regions naturally acquire antibodies to multiple rosetting parasite strains. Archived plasma samples from 40 adults and 40 children were tested for IgG recognition of the surface of live infected erythrocytes by flow cytometry, using four rosetting IgM Fc-binding parasite strains (Table 1). Because of spontaneous *var* gene switching (31, 37), cultured parasites contain a heterogeneous mix of infected erythrocytes expressing different PfEMP1 types, even after selection for rosetting. Therefore, we used a dual human plasma/rabbit anti-PfEMP1 surface staining protocol to allow specific examination of human IgG binding to infected erythrocytes expressing the rosette-mediating PfEMP1 variants of interest. For each parasite strain, the PfEMP1-positive infected erythrocytes were identified using rabbit polyclonal IgG against the rosette-mediating PfEMP1 N-terminal domain and an Alexa Fluor™ 647-conjugated anti-rabbit IgG secondary antibody. The human antibodies bound to this infected erythrocyte population were detected with an Alexa Fluor™ 488-conjugated anti-human gamma chain-specific secondary antibody (Figure S1). Any background staining of infected or uninfected erythrocytes, which occurs commonly with human sera and plasma (38), was corrected for as described in the methods and Figure S2. Examples of human plasma samples showing negative or strong positive IgG staining are shown in Figure S3.

**Figure 1:**
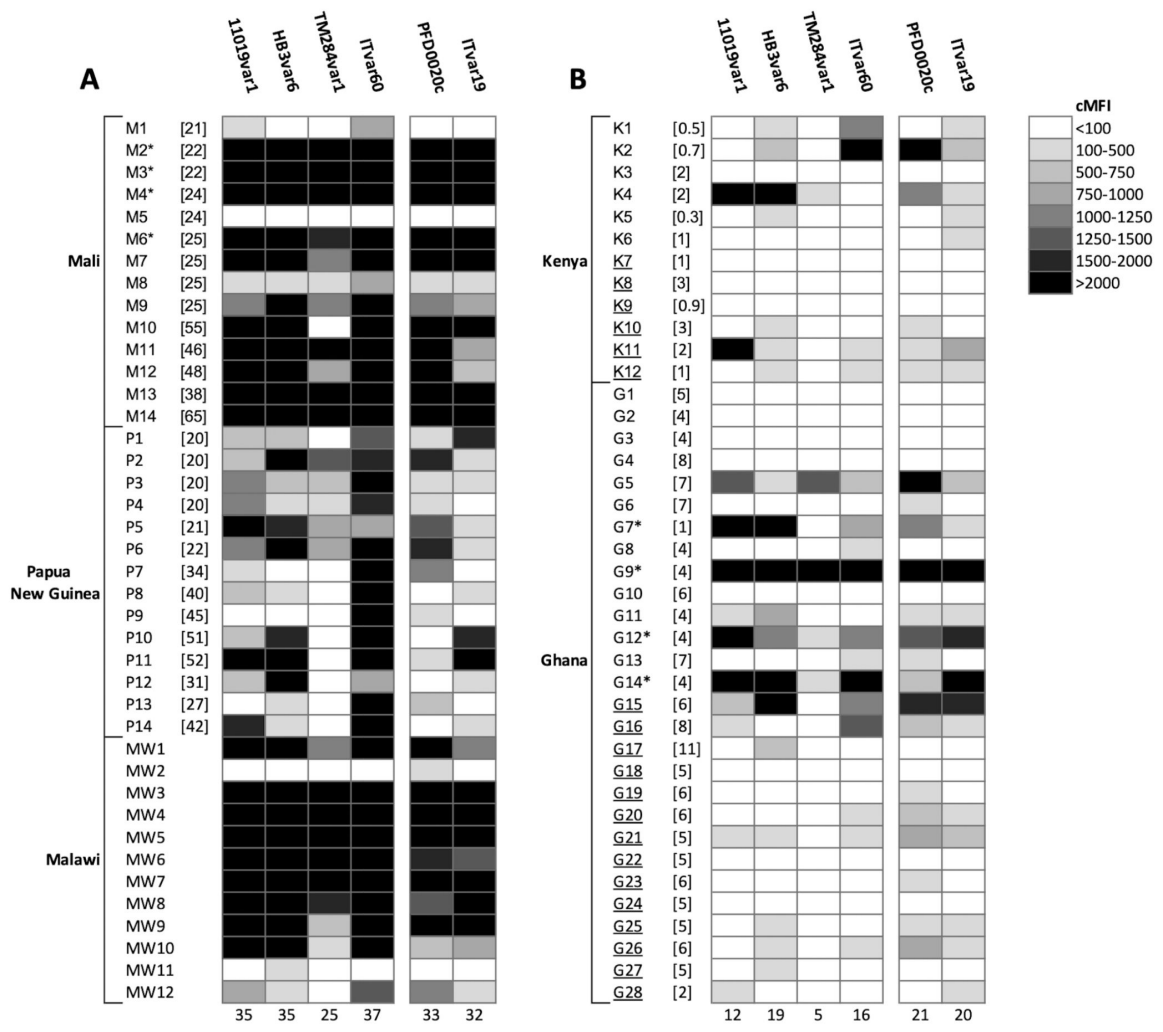
Recognition of infected erythrocytes expressing rosetting or DC8 PfEMP1 variants by human IgG. A) Adults’ plasma samples. B) Childrens’ plasma samples. Four rosetting parasite strains (expressing the PfEMP1 variants 11019VAR1, HB3VAR6, TM284VAR1 and ITVAR60) and two DC8-expressing human brain endothelial cell-binding parasite strains (expressing 3D7_PFD0020c and ITVAR19) were incubated with human plasma at 1/10 dilution. The infected erythrocytes expressing the specific variant of interest were detected with rabbit anti-PfEMP1 IgG as described in Figure S1, and the human IgG bound to those cells was detected with an Alexa Fluor™ 488-conjugated anti-human IgG (gamma chain) antibody at 1/1000 dilution. The corrected median fluorescence intensity (cMFI) was determined by subtracting non-specific background staining as described in the methods and Figure S2. Each plasma sample was tested in duplicate in each experiment, and the mean of the corrected MFI from two independent experiments for each parasite strain is shown. The number of positive plasma samples (cMFI >100) for each parasite strain is shown at the base of each column. The age in years of each individual at the time of plasma donation is shown in square brackets, if known. Samples from children with severe malaria are underlined. *Samples used in eluted antibody experiments.

The four rosetting parasite strains, which originated from Kenya, Honduras, Thailand and SE Asia, were well-recognised by adult plasma samples from Mali, Papua New Guinea and Malawi (Figure 1A). Seroprevalence values, defined here as the percentage of plasma samples positive for recognition of each strain, were calculated and 95% (38/40) of the adult samples recognised at least one rosetting strain, with 62.5% (25/40) recognising all four rosetting strains. For the childrens’ plasma samples, which came from donors in Kenya and Ghana aged 4 months to 11 years, 60% (24/40) recognised at least one rosetting strain, but only 4/40 (10%) recognised all four rosetting strains (Figure 1B). For comparison, IgG responses to two *P. falciparum* strains previously selected for binding to human brain endothelial cells, which express PfEMP1 variants associated with virulence, composed of a characteristic set of tandem adhesion domains known as “domain cassette 8” (DC8) (33), were tested for recognition with the same plasma set (Figure 1). 92.5% (37/40) of adults’ samples and 62.5% (25/40) of childrens’ samples recognised at least one of the DC8-expressing parasite strains, and 70% (28/40) of adults and 40% (16/40) of children recognised both strains.

### Detection of strain-transcending activity against rosetting strains using eluted antibodies

**Figure 2:**
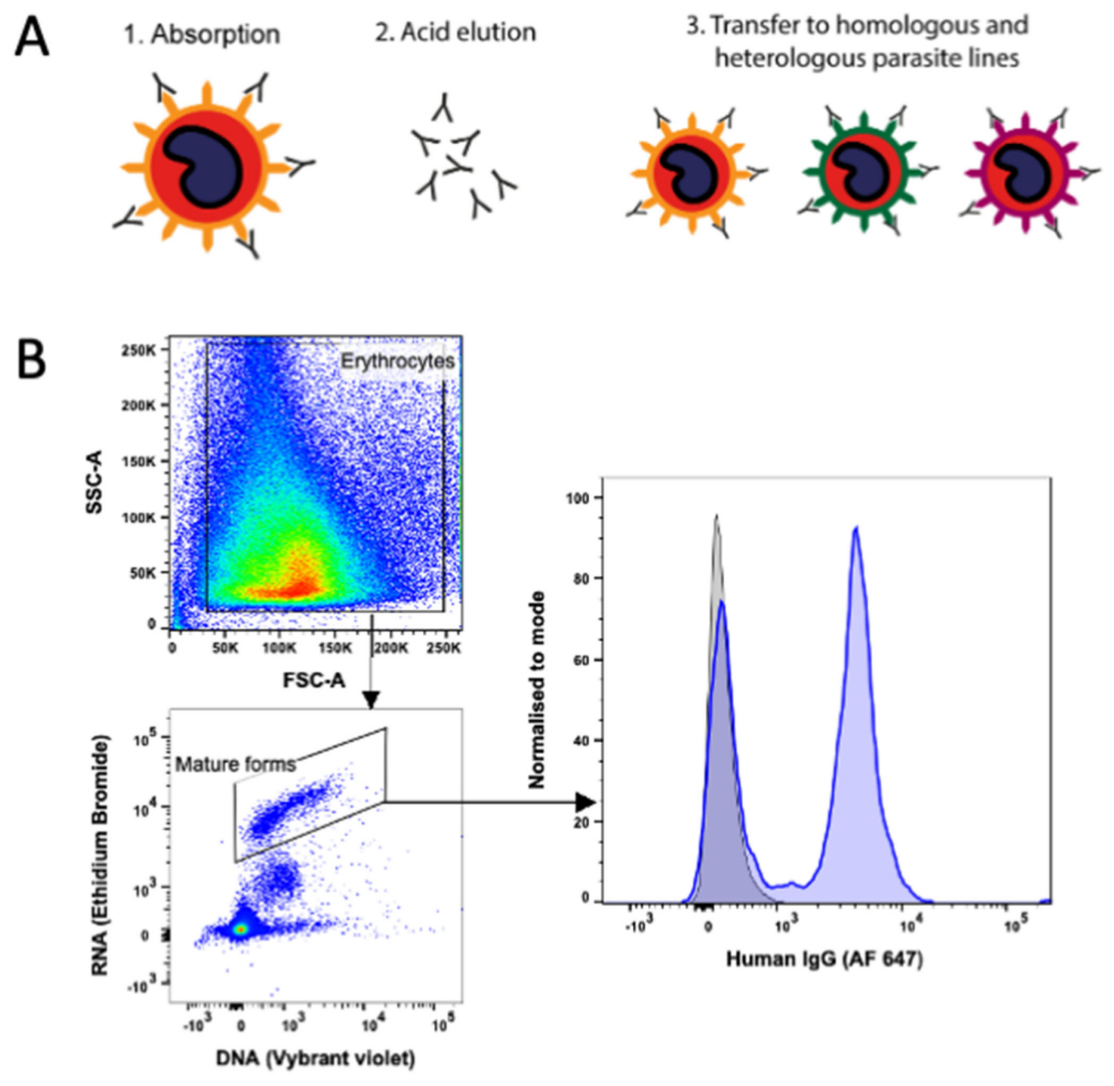
Method of detection of strain-transcending IgG in eluates. (A) Schematic diagram of the acid elution method. (B) Successful detection of IgG in eluates. Left, dot-plots showing the gating strategy used to identify mature infected erythrocytes in whole cultures. Forward and side scatter were used to exclude debris and set a gate on all erythrocytes, then mature pigmented-trophozoite and schizont infected erythrocytes were identified using Vybrant™ Dyecycle™ Violet (DNA stain, 1/2500 dilution) and Ethidium Bromide (DNA/RNA stain, 20 µg/ml) as the DNA and RNA high population (40). Right, fluorescence intensity histogram of ITvar60R+ mature infected erythrocytes stained with neat eluate of plasma M2 from ITvar60R+ (blue), compared to a concentration-matched human IgG control (grey). Bound human IgG was detected with an Alexa Fluor™ 647-conjugated anti-human IgG (gamma chain) antibody at 1/1000 dilution. A bimodal staining pattern is expected if the eluted antibodies recognise a VSA such as PfEMP1, which is expressed by some but not all of the infected cells in the culture.

Plasma from four adults (M2, M3, M4 and M6) and four children (G7, G9, G12, and G14) were chosen for eluted antibody experiments, on the basis of broad recognition (positive with 3 or 4 rosetting strains) and plasma availability. Each individual plasma sample was incubated with purified infected erythrocytes and the bound immunoglobulin was eluted from the infected erythrocyte surface using cold acid-elution (19, 39). ITvar60R+ and TM284R+ were selected as the adsorbing parasite strains due to the relative ease of growing large culture volumes and maintaining the rosetting phenotype in these strains. The eluates were tested for their ability to stain homologous and heterologous live infected erythrocytes as shown in Figure 2A. As the IgG in the eluates was present at low concentrations, co-staining with rabbit IgG to the PfEMP1 variant of interest was not included in the staining strategy in this case, to prevent the possibility of the rabbit IgG competing with the human IgG for PfEMP1. Instead, the mature PfEMP1-expressing infected erythrocyte population was identified by staining with both Vybrant™ DyeCycle™ Violet and Ethidium Bromide as previously described (40) (Figure 2B), and the human IgG median fluorescence intensity values were corrected for non-specific background staining as described in the methods.

**Figure 3:**
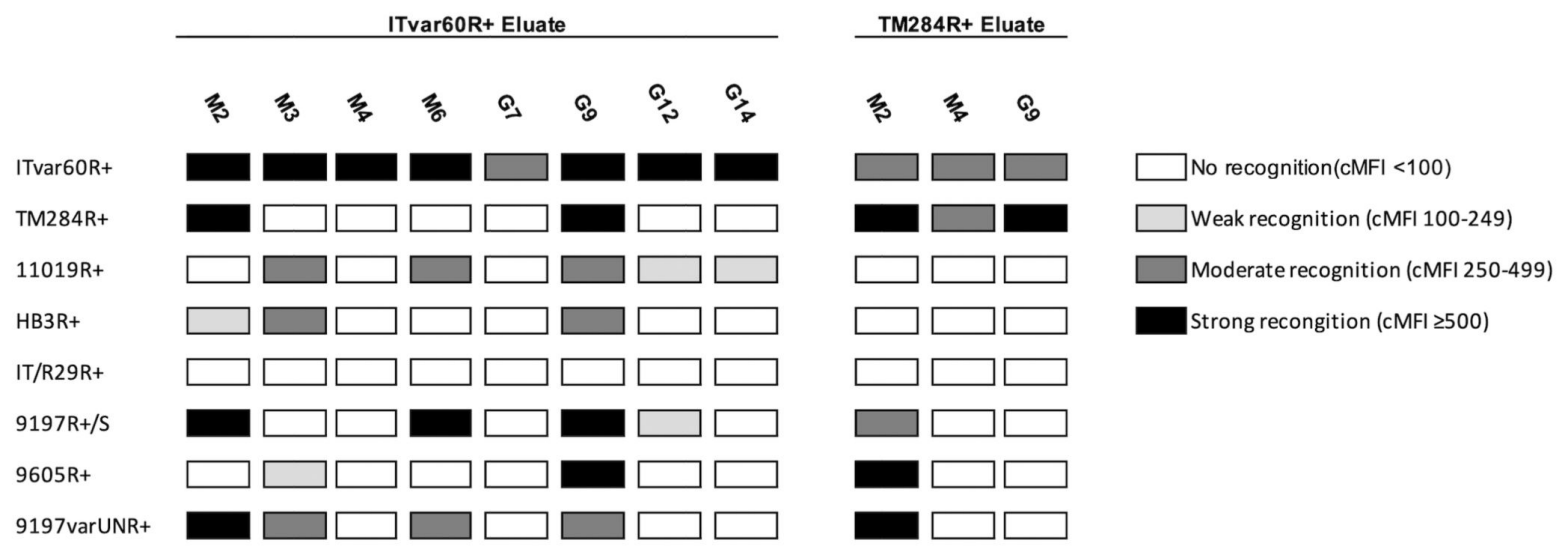
Summary of recognition of homologous and heterologous rosetting infected erythrocytes by eluted antibodies using flow cytometry. Eight rosetting parasite strains were stained with antibodies eluted from ITvar60R+ (8 individual donors; 4 adults M2, M3, M4, M6 and 4 children G7, G9, G12, G14) or from TM284R+ (3 individual donors; 2 adults M2 and M4 and 1 child G9). Mature infected erythrocytes were identified using Vybrant™ Dyecycle™ Violet at 1/2500 dilution (DNA stain) and Ethidium Bromide at 20µg/ml (DNA/RNA stain) (40). Eluates were used neat, and human IgG bound to mature infected erythrocytes was detected with an Alexa Fluor™ 647-conjugated anti-human IgG (gamma chain) antibody at 1/1000 dilution. Corrected median fluorescence intensities (cMFI) were adjusted for background staining as described in the methods. Due to the limited availability of eluate, the experiments were performed once.

Using ITvar60R+ as the adsorbing strain, all eight eluates recognised the homologous parasites, showing successful elution of IgG against the infected erythrocyte surface (Figure 2B, Figure 3 and Figure S4). For TM284R+, five eluates did not stain homologous parasites, therefore these samples were excluded, as this indicated failure to elute sufficient IgG for detection. The other three positive TM284R+ eluates and the eight positive ITvar60R+ eluates were used to stain geographically diverse rosetting strains expressing distinct PfEMP1 variants (Table 1), to look for strain-transcending activity. Nine of the eleven eluates had heterologous activity against at least one other rosetting strain, with six of these reacting with multiple heterologous strains (Figure 3 and Figure S4). The strain-transcending activity of eluates from ITvar60R+ was most frequent against 11019R+, with five of the eight eluates recognising this strain. The other rosetting strains were recognised by between two and four of the ITvar60R+ eluates, except for rosetting strain IT/R29R+, which was not recognised. Given that ITvar60R+ and IT/R29R+ are different variants from the same parasite genotype (IT/FCR3), it is perhaps not surprising that they are antigenically distinct, because shared epitopes within the same repertoire would reduce the number of immunologically novel variants available to facilitate parasite survival and prolong infection in the human host. For the TM284R+ eluates, strain-transcending activity against ITvar60R+ was most frequent, as all three eluates recognised this rosetting strain. The relatively high sequence similarity between the N-terminal DBLα domain (63% amino acid identity) and Cysteine-Rich Interdomain Region (CIDRβ, 82% amino acid identity) of these two variants has been noted previously (7).

The pattern of staining with the eluted antibodies allowed us to draw inferences about the nature of the conserved determinant(s) on the surface of infected erythrocytes. The entire mature-infected erythrocyte population was examined, which (as described above) consists of a mixture of cells expressing the rosetting PfEMP1 variant of interest and cells that have switched to express other PfEMP1 types. If the eluted antibodies were specifically recognising the rosette-mediating PfEMP1 variant-expressing cells, then some but not all infected erythrocytes should be recognised, resulting in a bimodal distribution on the fluorescence intensity histograms. If, on the other hand, the eluted antibodies were recognising a conserved epitope or protein present on all infected erythrocytes, then a single population of positively stained infected cells would be seen. Examination of the data shows a clear bimodal pattern of IgG reactivity in many cases, as would be expected for antibodies against a VSA such as PfEMP1 (Figure S4).

### Eluate M2(ITvar60R+) can disrupt rosettes

**Figure 4:**
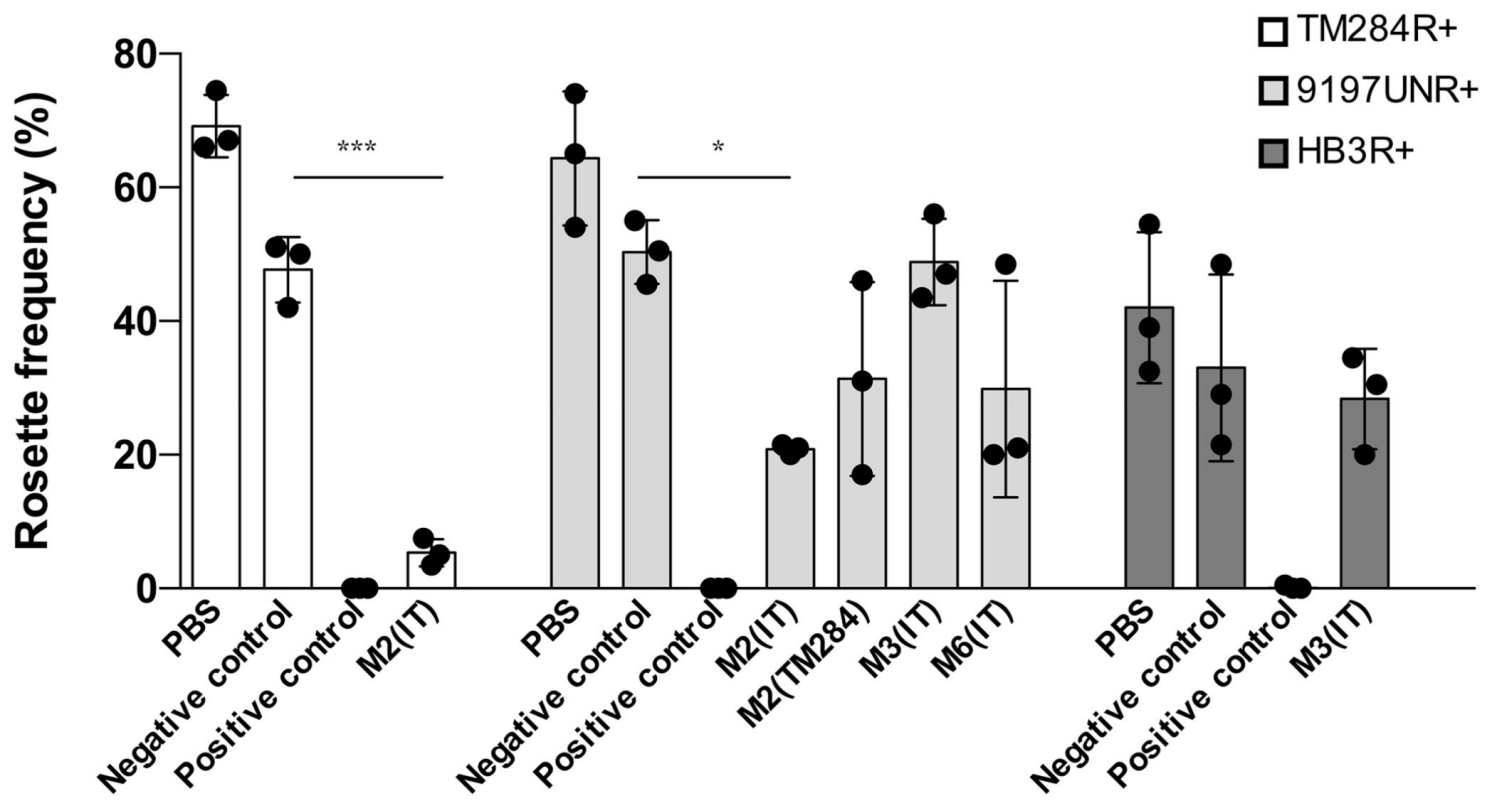
Rosette disrupting activity of eluates. Rosette frequency of parasite strains TM284R+, 9197UNR+ and HB3R+ (indicated in key) incubated in the presence of a negative control eluate that does not recognise mature infected erythrocytes, a positive control (antisera to the relevant PfEMP1 DBLα domain or heparin), or selected eluates which showed surface recognition against the tested parasite strain. The plasma and eluting strain for each eluate is indicated on the X axis. Results from three independent experiments are shown as individual data points. Bar heights show the mean of the three experiments, and error bars indicate the SD. Significant differences between the negative control and tested eluate by student’s t-test or one way ANOVA are shown, ***P<0.001, *P<0.05. All differences between the negative and positive controls were statistically significant (P<0.0001).

As well as being an immune target, PfEMP1 is the adhesion molecule that mediates rosetting in many *P. falciparum* strains (3–7). We therefore investigated whether the eluted antibodies had rosette-disrupting activity against heterologous strains, as this might be detected if they are targeting PfEMP1. Due to limited eluate availability, only some eluate/parasite strain combinations were tested. Eluate M4 (eluted from ITvar60) was used as a negative control, as it only recognised the homologous strain by flow cytometry, so it should not show any heterologous rosette-disrupting activity. Rabbit IgG raised to the DBLα domain of the rosette-mediating PfEMP1 variants for each strain was used as a positive control for rosette disruption, except for strain 9197varUNR+, where heparin was used. When incubated at a final dilution of 1 in 2, eluate M2 (eluted from ITvar60R+) disrupted rosettes in both TM284R+ and 9197UNR+ (Figure 4). This suggests that in the M2 plasma sample (eluted from IT parasites), PfEMP1 could be the target of the strain-transcending antibodies. The other eluates tested did not have statistically significant rosette disrupting activity on the parasite strains tested, either because they recognise epitopes or antigens distinct from the rosetting binding site, or because the concentration of IgG was too low to disrupt rosettes.

## Discussion

Here we have shown that both adults and children living in *P. falciparum* endemic regions have naturally acquired IgG responses to allopatric rosetting parasite strains. In adults, these responses were common, with seroprevalence ranging from 63%-93%, whereas in children the seroprevalence was lower, ranging from 13%-48%. These findings build on previous studies that have described the age-related build-up of anti-rosetting immunity and a high seroprevalence in adults of antibodies that recognise rosetting parasite strains (4, 6, 20, 41, 42). The results imply that *P. falciparum* isolates that share antigenic determinants with the culture-adapted rosetting strains used in this study circulate in the regions from which the plasma samples were derived, and that exposure to such isolates induces antibodies in the majority of the adult population. Given the diverse geographical origins of the plasma and parasite strains tested, this is in keeping with the existence of globally conserved epitopes on the surface of rosetting infected erythrocytes (7).

Using the eluted antibody technique, we went on to demonstrate the presence of strain-transcending antibodies in both adults and children that recognise the infected erythrocyte surface of genetically distinct rosetting strains. The use of acid elution to show the existence of strain-transcending antibodies against epitopes on live infected erythrocytes was first reported by Marsh and Howard in the 1980s (19). Surprisingly, in the four decades since then, this result has not to our knowledge been replicated until the work reported here. A more recent study by Tan et al. (24) used the mixed agglutination technique to show the existence of rare strain-transcending antibodies in Kenyan adults, which target some RIFIN family members. Mixed agglutination relies on the ability of bivalent IgG to cause mixed colour agglutinates if surface epitopes are shared between genetically distinct infected erythrocytes pre-stained with different colours (43). Another previous study using mixed agglutination sought to identify strain-transcending antibodies to three rosetting *P. falciparum* strains, Palo Alto VarO, IT4 R29 and 3D7 PF13 in Senegalese sera, but found only variant-specific responses (6). However, a study in Pakistan identified agglutinating and rosette-disrupting responses to heterologous parasites in convalescent serum from children recovering from malaria, suggesting the presence of strain-transcending responses to rosetting strains (44). Our results unequivocally demonstrate that human IgG eluted from the infected erythrocyte surface of one rosetting strain can recognise multiple heterologous rosetting strains, and therefore prove the existence of naturally acquired strain-transcending antibodies to rosetting infected erythrocytes in humans. This in turn implies the presence of conserved epitopes on infected erythrocyte surface antigens displayed by rosetting parasite strains.

Current evidence for the commonness of strain-transcending antibodies against *P. falciparum* VSAs is contradictory. Most previous studies using mixed agglutination to look for strain-transcending antibodies to the surface of infected erythrocytes indicate that strain-transcending activity is rarely detected in plasma samples from malaria endemic regions (24, 43, 45). Similarly, an extensive competition ELISA suggested that strain-transcending activity to PfEMP1 was very rare, only being detected in 1% of the 1245 heterologous protein combinations tested (46). In contrast, one study detected frequent mixed strain agglutinates in the presence of plasma from malaria-exposed Indian donors (47), and serological studies have demonstrated recognition of allopatric isolates, with which the donors could never have been infected (16, 19). Furthermore, people who have only ever been exposed to a single infecting genotype, such as tourists returning from malaria endemic areas, or participants in experimental malaria challenge studies, have been reported to develop antibodies to heterologous *P. falciparum* strains, or recombinant PfEMP1 domains from heterologous infected erythrocytes (48–50).

Some of the contradictory findings from prior studies may be explained if epitopes are only shared between functionally related PfEMP1 variants. In such a scenario, the choice of parasite strains examined in any study is crucial, and strain-transcending activity will only be detected if parasite strains expressing functionally related subsets of PfEMP1 are used. Prior studies have shown strain-transcending activity of human mAbs against the VAR2CSA PfEMP1 variant, which is responsible for infected erythrocyte adhesion to Chondroitin Sulfate A (CSA) in the placenta during pregnancy malaria (21, 22). These mAbs recognise the infected erythrocyte surface of VAR2CSA-expressing culture-adapted parasite strains and clinical isolates, but do not recognise parasite isolates expressing other PfEMP1 variant types. Our own prior work shows that antibodies generated in rabbits against some rosette-mediating PfEMP1 variants have strain-transcending activity against culture-adapted parasite strains and clinical isolates, but only against parasites with same dual rosetting IgM Fc-binding phenotype (7). Strain-transcending activity has also been described for antibodies against parasite strains showing adhesion to ICAM-1 (51) and EPCR (52), although much of the data for these functionally related subsets is based on recombinant protein assays. This is important because for PfEMP1, recognition and cross-reactivity of antibodies in ELISA-type assays using recombinant protein does not necessarily reflect recognition of native PfEMP1 on the infected erythrocyte surface (6)(7)(53). Hence, flow cytometry, agglutination assays or other functional experiments with live infected erythrocytes are needed to prove the presence of strain-transcending antibodies against native surface antigens.

Although the target of the strain-transcending antibodies described here is unknown and will require further investigation, we provide preliminary evidence that the rosette-mediating adhesin PfEMP1 may be the target of antibodies in at least one of the eluates, because the eluted IgG was able to disrupt the rosettes in two heterologous parasite strains. It is possible that the targets of the strain-transcending antibodies identified in this work are antigens other than PfEMP1. Recently, Tan and colleagues reported the existence of broadly strain-transcending antibodies to the surface of infected erythrocytes which recognised RIFINs (24). However, the strain-transcending activity observed here is unlikely to be due to the LAIR-1 insertions described by Tan et al., as such antibodies were rare, being found in only 3 out of 557 Kenyan adult plasma examined (24).

The work presented here has several limitations. In the eluted antibody experiments, strain-transcending activity could be missed due to incomplete elution of IgG from the surface of the adsorbing infected erythrocytes, which occurred commonly (data not shown). Furthermore, low concentrations of strain-transcending antibodies in plasma would be hard to detect using our methods, and our approach does not identify the target(s) of strain-transcending antibodies. This study does, however, confirm the biological relevance of strain-transcending antibodies, and by using live infected erythrocytes rather than recombinant proteins, it avoids the problems associated with serological assays in which recognition of recombinant PfEMP1 domains does not necessarily indicate recognition of native protein (53).

Despite the limitations of our study, the finding of high seroprevalence against allopatric rosetting isolates in adults from malaria-endemic regions, along with the demonstration of relatively common strain-transcending antibodies to the rosetting infected erythrocyte surface, are encouraging findings that warrant further investigation. Future experiments will aim to identify the targets of strain-transcending antibodies on the infected erythrocyte surface, and to characterise the conserved epitopes that are recognised. Identification of cryptic conserved infected erythrocyte surface epitopes may be a useful approach for the development of interventions against severe malaria (54). For example, monoclonal antibodies that reverse rosetting could be a useful adjunctive therapy for severe malaria, or antigens containing conserved epitopes recognised by strain-transcending antibodies could be part of a vaccine to prevent severe disease (16). Similar approaches based on non-rosetting virulence mechanisms, such DC8-associated adhesion (55), could generate a cocktail aimed at reversing or preventing dense sequestration in vital organs and reducing death and disability from malaria.

## Materials and Methods

### Human plasma samples and research ethics

80 anonymised archived human plasma samples from residents of malaria-endemic regions (40 adults and 40 children) were used. The samples were collected after informed consent from donors or their parents/guardians, as part of previous studies in Mali (56), Malawi (57), Papua New Guinea (58), Kenya (59), and Ghana (Y. Azasi, G. Awandare and J.A. Rowe, unpublished). The number of samples studied from each location was Mali (n=14), Malawi (n=12), Papua New Guinea (n=14), Kenya (n=12) and Ghana (n=28); the samples were selected randomly from those available from the above prior studies. The adults were healthy and/or asymptomatic whereas the children had clinical *P. falciparum* infection at the time of plasma collection. The negative control human serum pool was made from 15 European donors at the Scottish National Blood Transfusion Service (SNBTS), Edinburgh, UK. Ethical approval was granted as described (56–59) and from the University of Edinburgh, School of Biological Sciences Ethical Review Panel (arowe002), Scottish National Blood Transfusion Service (SNBTS) Ethics Review Board (19~6), Ghana Health Service Review Committee ID No. GHS-ERC: 03/05/14 and Noguchi Memorial Institute for Medical Research-IRB study# 020/13-14.

### Rabbit antibodies to PfEMP1

The antibodies to specific PfEMP1 variants were generated as described previously by immunising rabbits with recombinant DBLα protein produced in *E. coli*, then purifying total IgG from the rabbit serum on a protein A column (7)(33)(J.A. Rowe, unpublished data).

### *P. falciparum* strains

The geographical origin, *var* gene/PfEMP1 expression and adhesion phenotype of the culture-adapted parasite strains studied are shown in Table 1. In addition to the well-established laboratory strains IT, HB3, 3D7 and TM284, three recent culture-adapted Kenyan strains (24) were used. One of the Kenyan strains (11019R+) spontaneously showed high levels of rosetting, and the other two (9197 and 9605) were selected for rosetting using Percoll or gelatin sedimentation (60). The strain 9197R+ showed two distinct phenotypic forms in parallel selections, 9197R+ which expresses the rosette-mediating PfEMP1 variant 9197varR1 (encoded by *PC0053-C.g687* in the Pf3k database (61)) (J.A. Rowe, unpublished data) and 9197varUNR+ which does not express 9197varR1, and whose predominant PfEMP1 type has not yet been identified.

### *P. falciparum* culture

*P. falciparum* strains were cultured at 2% haematocrit in O+ erythrocytes and RPMI-1640 medium (Lonza, BE12-167F or Gibco, 31870-025) supplemented to give the following final concentrations: L-glutamine 2 mM (Gibco, 2530-081), glucose 16 mM, 4-(2-hydroxyethyl)-1-piperazineethanesulfonic acid (HEPES) 25 mM (Lonza, 17-737F), gentamicin 25 μg/ml (Lonza, 17-518L), AlbuMAX II Lipid-rich bovine serum albumin (BSA) (Gibco 11021037) 0.25%, and pooled human serum 5%, and the pH adjusted to 7.2-7.4 with sodium hydroxide. Both human serum and blood for culture were obtained from SNBTS, Edinburgh, UK. Cultures were gassed using 94% nitrogen, 1% oxygen and 5% carbon dioxide (BOC, 280648-L) and incubated at 37°C. Cultures were maintained at ~2% haematocrit and 2-10% total parasitaemia. Rosetting was maintained by selection with plasmagel flotation or Percoll selection approximately once a week (60).

### Immunofluorescent staining of live infected erythrocytes with human plasma

Human plasma samples were tested against four IgM-binding rosetting parasite strains (ITvar60R+, TM284R+, HB3R+ and 11019R+)(7) and two DC8-expressing parasite strains that were previously selected for binding to human brain endothelial cells (ITvar19 and 3D7_PFD0020c)(33). Parasite culture in PBS/1% BSA at 1% haematocrit (Ht) was incubated for 45 min at 37°C with 1/10 dilution of human plasma from malaria-endemic region donors (or non-endemic European serum pool as a negative control) and 20 µg/ml of rabbit IgG raised against the DBLα region of the rosette-mediating PfEMP1 variant for each parasite strain (7)(or non-immunised rabbit IgG as a negative control). After washing three times with PBS the cells were resuspended in PBS/1% BSA with 1/2500 dilution of Vybrant™ DyeCycle™ Violet (Invitrogen, V35003), 1/1000 Alexa Fluor™ 647-conjugated cross-absorbed goat anti-rabbit IgG (Invitrogen, A-21244) and 1/1000 Alexa Fluor™ 488-conjugated cross-absorbed goat anti-human IgG (heavy chain specific) (Biotium, 20444). The plates were incubated for 30 min at 37°C in the dark then washed twice with PBS and once with PBS/1% BSA. After the final wash, cells were fixed with 0.5% paraformaldehyde (Thermo Fisher, 28906) in PBS for 15 min at room temperature. The paraformaldehyde was removed and the cells resuspended in PBS/0.1% BSA/0.01% sodium azide with 100 µg/ml fucoidan (Molekula, 9072-19-9) added to disrupt rosettes (7). The plates were protected from light and stored at 4°C until analysis by flow cytometry within 24 h.

#### Data acquisition and gating strategy for human plasma experiments

The 96 well plates were read on a BD LSRFortessa™ (BD Biosciences) at a flow rate of 2 µl/s until a maximum of 500,000 events were recorded, or 50s had elapsed, whichever came first. The data were analysed in FlowJo v.10 (BD Biosciences). All events were gated using forward and side scatter to exclude debris, followed by gating on all infected erythrocytes (Vybrant™ DyeCycle™ Violet high), followed by PfEMP1 positive cells (Alexa Fluor™ 647 high), to identify the rosetting PfEMP1 variant-expressing infected erythrocyte cell population of interest. Human IgG bound to these cells was detected in the 488 channel and the median fluorescence intensity (MFI) was recorded. An example of the gating strategy is shown in Figure S1.

#### Data analysis and visualisation for human plasma experiments

Because human plasma/sera sometimes show non-specific binding to infected and/or uninfected erythrocytes, controls were carried out and fluorescence intensity measurements were corrected for background staining (cMFI) as described (38). The cMFI = (MFI of the PfEMP1-positive infected erythrocytes stained with endemic plasma – MFI of the uninfected erythrocytes from the same sample) - (MFI of the PfEMP1-positive infected erythrocytes stained with the European negative control serum pool – MFI of the uninfected erythrocytes from the same sample). An example is shown in Figure S2. To visualise the data, the cMFI values for all plasma samples against all parasite strains were summarised in a checkerboard. To quantify the seroprevalence (percentage of plasma samples giving a positive result) for each parasite strain, a mean cMFI of >100 fluorescence units (fu) from two independent experiments was considered positive. All cMFI values below 100 fu were considered negative, as recognition was not compelling when the cMFI was <100 (Figure S3). This additional cut-off was applied to reduce the incidence of type 1 error.

### Magnetic-activated cell sorting (MACS) to purify infected erythrocytes

Prior to adsorption with human plasma, infected erythrocytes of strains ITvar60R+ or TM284R+ were purified by MACS as described previously (39), with the addition of either 1 mg/ml heparin (Sigma-Aldrich, H4784-1G) for ITvar60R+ or 50 μg/ml fucoidan (Molekula, 9072-19-9) for TM284R+, to disrupt rosettes. Eluted infected erythrocytes were washed twice to remove heparin or fucoidan then resuspended in RPMI 1640 containing 2 mM L-glutamine and 25mM HEPES, but lacking bicarbonate (Gibco, 13018-015), supplemented to 16 mM glucose and 25 μg/ml gentamicin and adjusted to pH 7.2-7.4 with sodium hydroxide to make incomplete binding medium (ICBM). 20-50 μl of rosetting infected erythrocytes were obtained, with a parasitaemia of at least 60-70%.

### Adsorption and antibody elution from the infected erythrocyte surface

Eight individual plasma samples (M2, M3, M4, M6, G7, G9, G12, G14) showing broad recognition of rosetting strains were used in adsorption and antibody elution experiments. Prior to adsorption, any antibodies recognising uninfected erythrocytes in each plasma sample were pre-adsorbed by incubation with an equal packed cell volume of O+ erythrocytes from a Scottish donor at 20 rpm on a rotating wheel for 1 hour at room temperature, and the process repeated with fresh erythrocytes a total of 4 times. 20-50 μl PCV of MACS-purified infected erythrocytes of strains ITvar60R+ or TM284R+ were resuspended in 100 μl of ice-cold pre-absorbed plasma and incubated on ice for 90 min with gentle re-suspension every 10 min. The cells were then washed five times in 1 ml of ice-cold 0.15 M NaCl then resuspended in 50 μl of ice-cold 0.15 M NaCl. Three rounds of elution were undertaken at successively lower pH points (39). First, 50 μl ice-cold glycine-HCl at pH 2.7 was added to the resuspended cells and placed on ice for 2 min. The cells were pelleted by centrifugation and the supernatant transferred to a fresh tube and neutralised with 3 μl 2M ice-cold Tris-base. After re-suspension of the cell pellet in 50 μl ice-cold 0.15M NaCl, the elution was repeated with 50μl glycine-HCl at pH 2.2, with the supernatant transferred into a tube containing 6μl 2M Tris-base. This was repeated once more with glycine-HCl at pH 1.7 and transfer of the supernatant into a tube containing 8 μl 2M Tris-base. The pH of the eluted fractions was checked and adjusted to pH 7 by the addition of further 2M Tris-base as required. All three eluted fractions were centrifuged at 9447 xg for 5 min to pellet debris and the three fractions pooled into a clean tube. The eluted antibody was buffer-exchanged into PBS using a Millipore Amicon ultra 100K microcentrifuge concentration unit (Merck, UFC510024) with four rounds of buffer-exchange using 450 µl of PBS per round. The purified eluate was recovered in 20 μl total volume and stored at 4°C until use. For most samples, eluate volume was adjusted with PBS to 150μl total volume to give sufficient volume for downstream experiments, resulting in protein concentrations up to 46 μg/ml. The exceptions were M2 eluates from both ITvar60R+ and TM284R+, which were adjusted to total volumes of 216 μl and 180 μl respectively. This was to bring their protein concentrations to below 200 μg/ml, which was the concentration used for the human IgG negative control (Sigma, I2511) in subsequent experiments. These diluted eluates were used neat in the staining experiments.

### Immunofluorescent staining of infected erythrocytes with eluted antibodies

Eight rosetting parasites strains (Table 1) were stained with eluted antibodies. Staining for recognition of the surface of live infected erythrocytes was carried out as described above for staining with human plasma, with modifications as follows: i) the culture suspension at 1% Ht was resuspended in neat eluate or negative control purified human IgG (Sigma, I2511) at 200 μg/ml in PBS ii) the samples were not assayed in duplicate due to limited eluate availability and each parasite strain was tested once. iii) the staining strategy did not include variant specific rabbit polyclonal IgG. Instead, the secondary antibody 1/1000 Alexa Fluor ™ 647-conjugated goat anti-human IgG (gamma chain specific) (Biotium, 20448) in PBS/1% BSA contained both 1/2500 of Vybrant™ DyeCycle™ Violet and 20 μg/ml Ethidium Bromide (Sigma, 46067) to allow gating on mature (pigmented trophozoite and schizont)-infected erythrocytes (40).

#### Data acquisition and gating strategy for eluted antibody experiments

Data acquisition was as described above for human plasma samples. All events were gated using forward and side scatter to exclude debris, followed by gating on mature infected erythrocytes (Vybrant™ DyeCycle™ Violet high, Ethidium bromide high). Human IgG bound to these cells was detected in the 647 channel and median fluorescence intensity recorded.

#### Data analysis and visualisation for eluted antibody experiments

For the eluted antibody experiments, cMFI = (MFI of the mature infected erythrocytes stained with eluted antibody - MFI of the uninfected erythrocytes from the same sample) - (MFI of mature infected erythrocytes stained with the commercial negative control human IgG – MFI of the uninfected erythrocytes from the same sample). To visualise the data, the cMFI values for each eluate against each parasite strain were summarised in a checkerboard.

### Rosette disruption with eluates

Parasite culture at ~2% Ht was stained with 25 μg/ml Ethidium Bromide for 5 min at 37°C. The supernatant was removed and the cells resuspended at 4-5% haematocrit in RPMI ICBM (as described above for magnetic-activated cell sorting) supplemented with 15% heat-inactivated normal human serum from Scottish donors. 2μl of PBS or eluate were added to 2μl aliquots of the pre-stained culture suspension and incubated for 30 min at 37°C with resuspension after 15 min. The “eluate negative” sample M4 (IT) as chosen as a negative control because this eluate did not recognise any heterologous parasites by flow cytometry, so would not be expected to disrupt rosettes in heterologous strains. As a positive control, 2 µl of 1mg/ml rabbit IgG against the specific PfEMP1 variant for each parasite strain (7) or 1mg/ml heparin (Sigma-Aldrich, H4784-1G) was added. The aliquot identities were masked before counting to prevent observer bias. Wet preparations were made on multi-spot slides and at least 200 mature infected erythrocytes per spot were observed using a Leica DM2000 fluorescence microscope and 40x objective, with simultaneous brightfield/fluorescence to visualise both infected and uninfected erythrocytes. The rosette frequency (percentage of mature infected erythrocytes binding two or more uninfected erythrocytes) was recorded. Three independent experiments were done for each parasite strain/eluate combination tested. Not all parasite strain/eluate combinations could be tested due to limited eluate availability. The mean rosette frequency in the presence of eluted antibody was compared to that in the M4 “eluate negative” control using Student’s t test or one-way ANOVA in GraphPad Prism v8.

## Supplementary Material

Figure S1-S4

## Figures and Tables

**Table 1:** Geographical origin and adhesion phenotype of the *P. falciparum* strains studied.

Genotype [alternative name] (reference)	Origin	Parasite strain [alternative names] (reference)	*Var* gene transcribed/DC* [alternative name] (reference)	Adhesion phenotype^$^
IT [IT4, IT04, FCR3] (28)	South-East Asia (29)	ITvar60R+ [R+PA1, PAR+, FCR3S1] (25, 30)	*ITvar60*/DC11 *[FCR3S1.2var2]* (5, 7)	IgM-positive rosetting (25, 26)
		IT/R29 [R29, R29R+](31) IT-HBEC (32) TM284R+ (25) HB3R+(7)	*ITvar9*/DC16 *[R29var1]* (3)	IgM-negative rosetting (26)
			*ITvar19*/DC8 (33)	HBEC^#^-binding (33)
TM284 (25)	Thailand (25)		*TM284var1*/DC11 (7, 27)	IgM-positive rosetting (25, 26)
HB3 (34, 35)	Honduras (34, 35)		*HB3var6*/DC11 (7)	IgM-positive rosetting (7)
11019 [PFKE10] (24)	Kenya (24)	11019R+	*11019varR1/DC11 [PX0203.g54)]* (UP)	IgM-positive rosetting (UP)
9197 [PC0053-C] (UP)	Kenya (24)	9197R+	*PC0053-C.g687* (UP)	IgM-negative rosetting (UP)
		9197varUNR+ 9605R+ 3D7-HBEC (32)	Unknown	
9605 [PFKE08] (24)	Kenya (24)		Unknown	IgM-negative rosetting (UP)
NF54 (36)	Africa (29)		*3D7_PFD0020c* (33)	HBEC^#^-binding (33)

* DC: domain cassette, a characteristic set of adhesion domains arranged in tandem, used to classify PfEMP1 variants into functionally-related types (17). The PfEMP1 architecture and amino acid sequence identities between domains for these variants are given in references (7) and (33).

$ parasite strains that have the dual rosetting and IgM Fc-binding phenotype are here designated “IgM-positive rosetting” and those that form rosettes but do not bind IgM-Fc are designated “IgM-negative rosetting”.

# HBEC: human brain endothelial cell

UP: Unpublished.

## Data Availability

All raw data are available from the authors on request.
