# Supplementary material for: Detection of naturally acquired, strain-transcending antibodies against rosetting Plasmodium falciparum strains in humans
Source: Infect Immun. Author manuscript; Available in PMC 2024 Aug 16. (PMC11238554; doi:10.1128/iai.00015-24)
Supplement: Figure S1-S4 [file EMS197799-supplement-Figure_S1_S4.pdf]

Supplementary figures

McLean *et al*. Detection of naturally acquired, strain-transcending antibodies against rosetting *Plasmodium falciparum* strains in humans

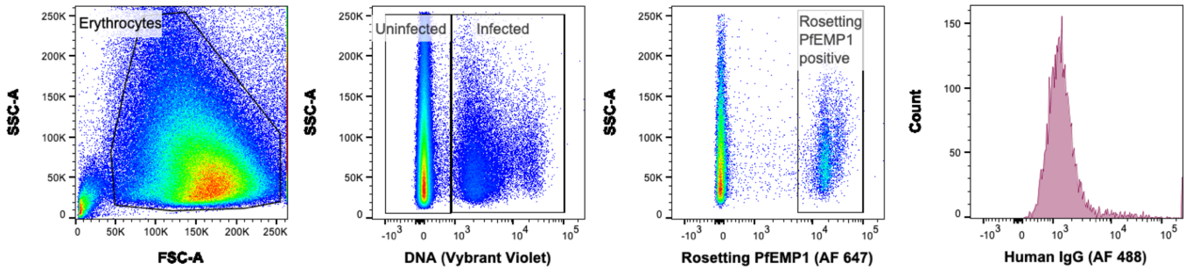

Figure S1. Gating strategy for detection of naturally acquired human IgG to rosetting parasite strains by flow cytometry. Forward and side scatter were used to exclude debris and gate on all erythrocytes (from left, first panel). All infected erythrocytes were identified by DNA staining with Vybrant™ Dyecycle™ Violet (second panel), and those that stained surface positive for the rosette-mediating PfEMP1 variant of interest were detected with 20 µg/ml rabbit polyclonal IgG against the NTS-DBLα domain of the PfEMP1 variant and an Alexa Fluor™ 647-conjugated anti-rabbit IgG secondary antibody at 1/1000 (third panel). The median fluorescence intensity (MFI) of the Alexa Fluor™ 488 channel (human IgG stain) for this “rosetting PfEMP1 positive” population was quantified (fourth panel). Human plasma was used at 1/10 dilution and Alexa Fluor™ 488-conjugated anti-human IgG (gamma chain) was used at 1/1000 dilution. AF, Alexa Fluor™.

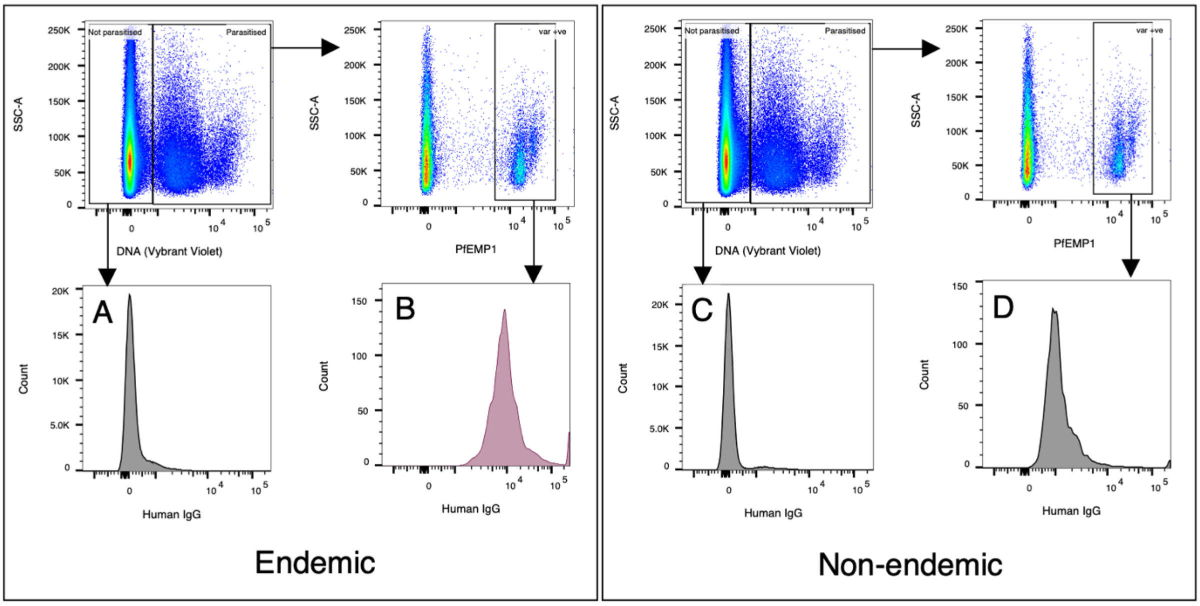

Figure S2. Correction of the Alexa Fluor^{TM} 488 median fluorescence intensity. Left panel, an example of gating and human IgG staining for a *P. falciparum* culture incubated with plasma (1/10 dilution) from a malaria-exposed individual ("Endemic"). Right panel, an example of gating and human IgG staining for the same *P. falciparum* culture incubated with 1/10 dilution of the negative control European serum pool ("Non-endemic"). The specific human IgG response is quantified by subtracting the Alexa Fluor^{TM} 488 MFI of the uninfected erythrocyte population (Vybrant^{TM} Dyecycle^{TM} Violet negative) (A) from the Alexa Fluor^{TM} 488 MFI of the infected erythrocyte population expressing the PfEMP1 variant of interest (B), which corrects for the presence of any anti-erythrocyte antibodies in the "endemic" sample. This is further corrected for any background staining observed with non-endemic negative control sera on infected erythrocytes expressing the PfEMP1 variant of interest (D), minus the MFI of uninfected erythrocytes with the negative control pool (C). Overall, this gives the cMFI = (B - A) - (D - C) [43]. The signal from the test plasma is shown in pink (B) and the negative control serum in grey (D). DNA staining was with 1/2500 dilution of Vybrant^{TM} Dyecycle^{TM} Violet (upper left panels). PfEMP1 was detected with 20µg/ml rabbit polyclonal IgG against the NTS-DBLα domain of the PfEMP1 variant of interest and an Alexa Fluor^{TM} 647-conjugated anti-rabbit IgG secondary antibody at 1/1000 (upper right panels). An Alexa Fluor^{TM} 488-conjugated anti-human IgG (gamma chain) antibody at 1/1000 dilution was used to detect human IgG (lower panels).

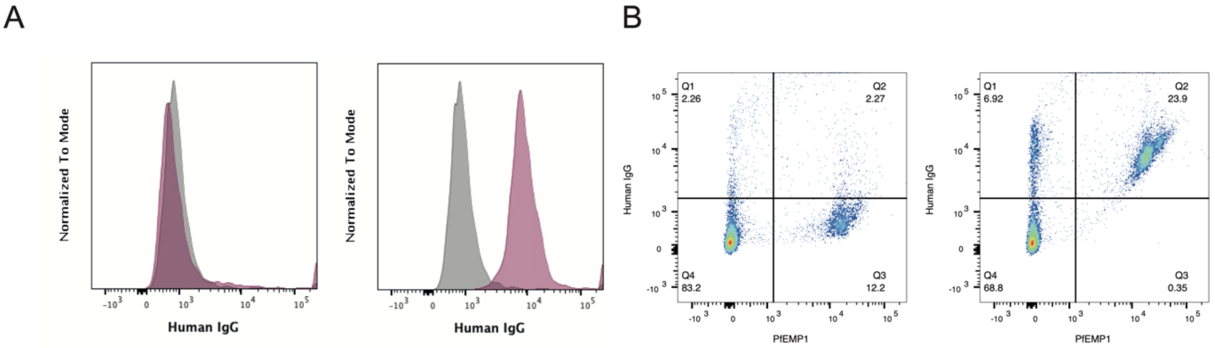

Figure S3. Examples of negative and positive human plasma staining of infected erythrocytes. A) Fluorescence intensity histograms of rosetting PfEMP1-positive cell populations exemplifying a plasma sample giving a negative result (plasma MW11 to parasite 11019R+, left), and a strong positive result (plasma M3 to parasite 11019R+, right). Red, endemic region plasma sample; grey, negative control European serum pool. B) Dot-plots of staining with the plasma samples shown in A, but rather than only rosetting PfEMP1-positive cells, all infected erythrocytes including ring-stages are shown. Only the PfEMP1 positive infected erythrocytes (right side quadrants) are used in the analysis. Human plasma and sera were used at 1/10 dilution. An Alexa Fluor™ 488-conjugated anti-human IgG (gamma chain) antibody was used at a 1/1000 dilution to detect human IgG. Surface staining for PfEMP1 was with 20 μg/ml rabbit polyclonal IgG raised against the DBLα domain of the PfEMP1 variant of interest and detected with an Alexa Fluor™ 647-conjugated anti-rabbit IgG secondary antibody at 1/1000 dilution.

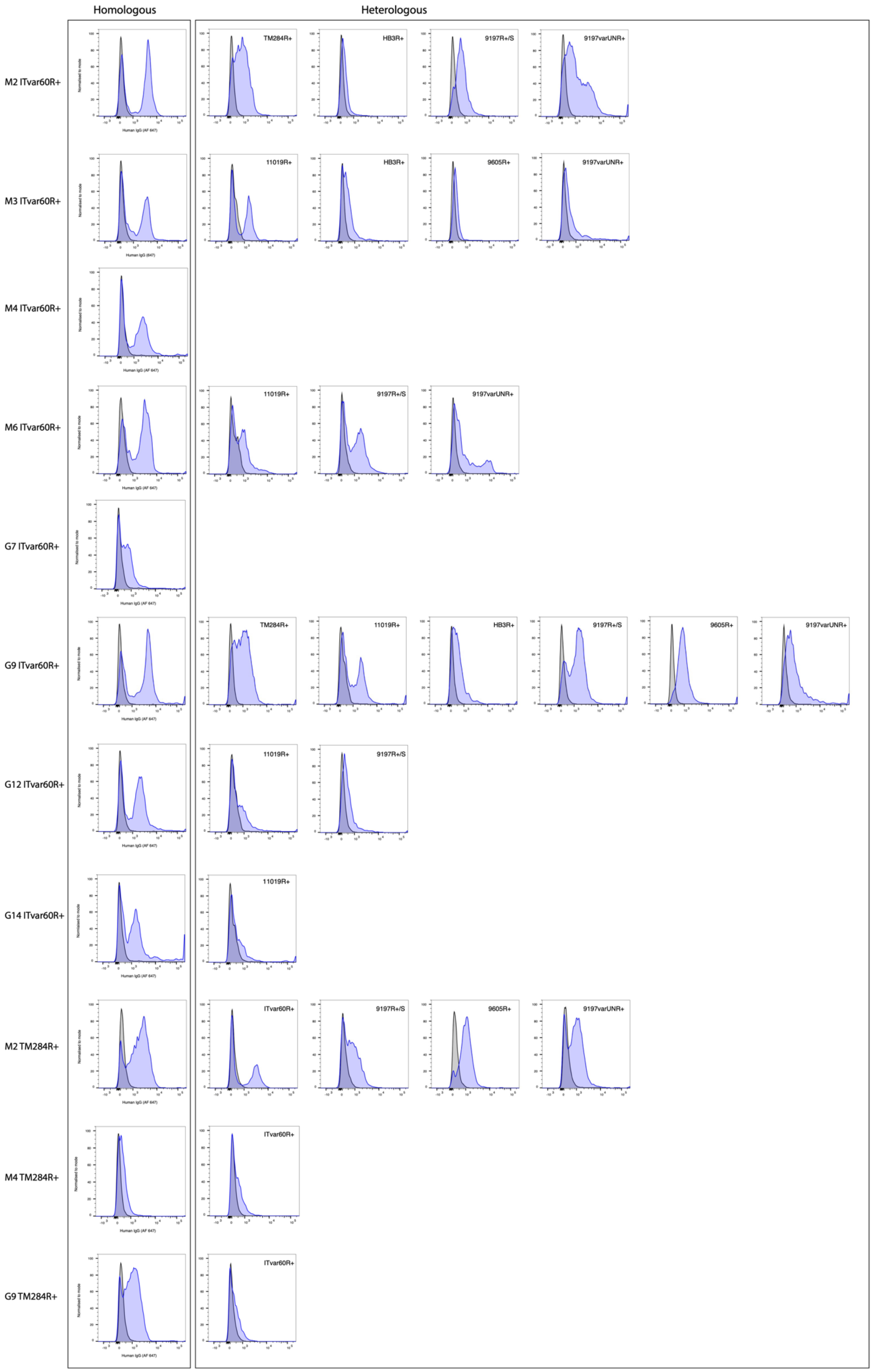

Homologous
Heterologous
M2 ITvar60R+
TM284R+
HB3R+
9197R+/S
9197varUNR+
M3 ITvar60R+
11019R+
HB3R+
9605R+
9197varUNR+
M4 ITvar60R+
M6 ITvar60R+
11019R+
9197R+/S
9197varUNR+
G7 ITvar60R+
G9 ITvar60R+
TM284R+
11019R+
HB3R+
9197R+/S
9605R+
9197varUNR+
G12 ITvar60R+
11019R+
9197R+/S
G14 ITvar60R+
11019R+
M2 TM284R+
ITvar60R+
9197R+/S
9605R+
9197varUNR+
M4 TM284R+
ITvar60R+
G9 TM284R+
ITvar60R+

Figure S4. Recognition of homologous and heterologous strains by eluted antibody. Fluorescence intensity histograms of mature infected erythrocytes, incubated with eluted antibody (blue) or a human IgG control (grey). Each row of plots shows the results for one eluate, with the plasma and adsorbing parasite strain indicated to the left of each row. "Homologous" shows the eluate tested against the parasite strain used for adsorption, whereas "heterologous" shows the eluate tested against other parasite genotypes, with the parasite strain name given in the top right corner of the histogram plot. Only positive (cMFI $\geq$100) results are shown. Mature infected erythrocytes were identified using Vybrant™ Dyecycle™ Violet at 1/2500 dilution (DNA stain) and Ethidium Bromide at 20μg/ml (DNA/RNA stain). Eluates were used neat, and human IgG bound to mature infected erythrocytes was detected with an Alexa Fluor™ 647-conjugated anti-human IgG (gamma chain) antibody at 1/1000 dilution.
